# Advances in our understanding of the molecular pathogenesis of necrotizing enterocolitis

**DOI:** 10.1186/s12887-022-03277-3

**Published:** 2022-04-25

**Authors:** Xue Cai, Alena Golubkova, Catherine J. Hunter

**Affiliations:** Division of Pediatric Surgery, Department of Surgery, University of Oklahoma Health Sciences Center, Oklahoma City, OK 73104 USA

**Keywords:** Necrotizing enterocolitis, Immature immune system, Neonatal inflammation, Epigenetic modification, Regulatory RNAs, Single nucleotide polymorphisms, Microvasculature

## Abstract

Necrotizing enterocolitis (NEC) is a multifactorial and complex disease. Our knowledge of the cellular and genetic basis of NEC have expanded considerably as new molecular mechanisms have been identified. This article will focus on the current understanding of the molecular pathogenesis of NEC with a focus on the inflammatory, immune, infectious, and genetic mechanisms that drive disease development.

## Background

**Fig. 1:**
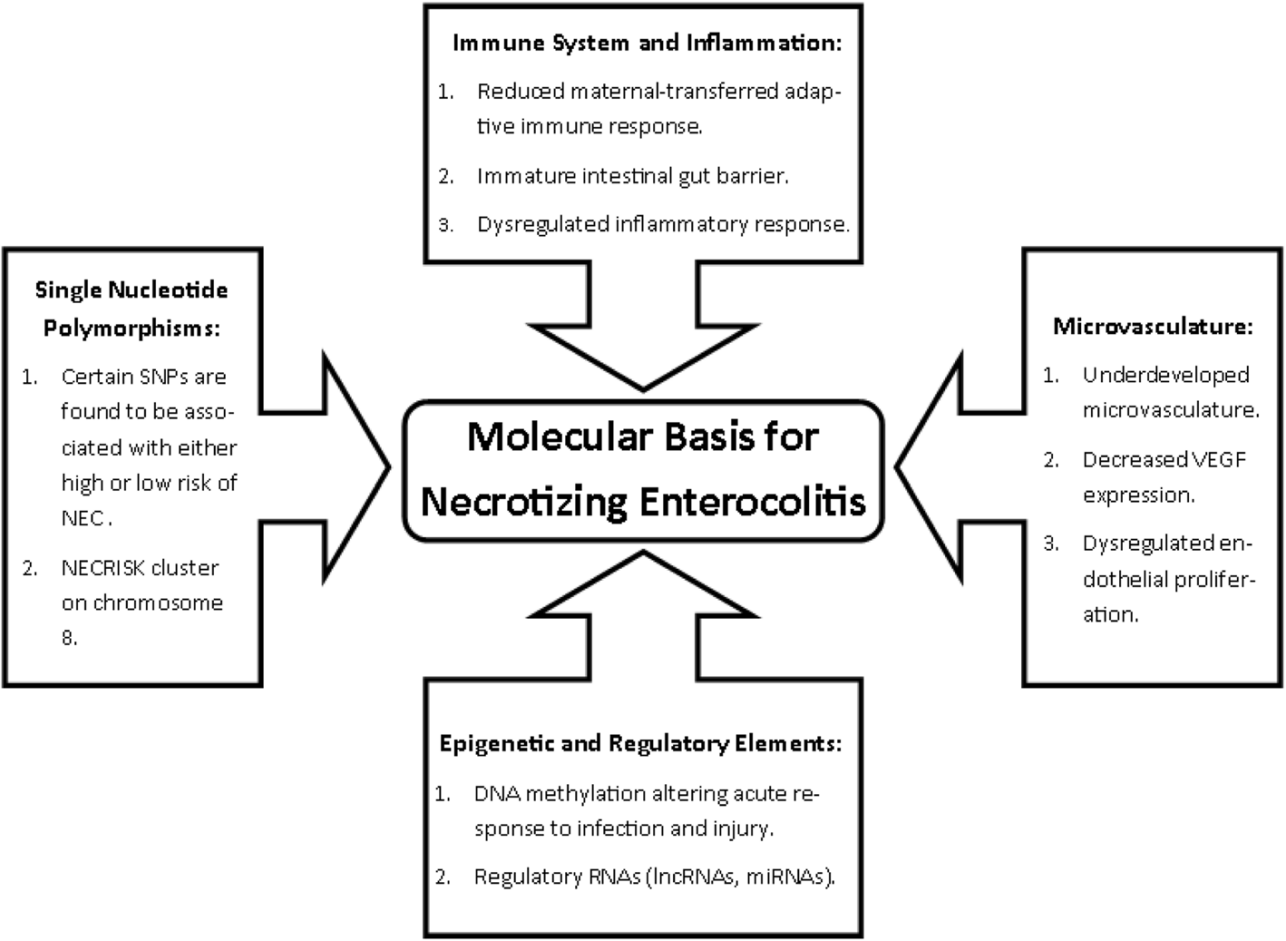
Summary of the current understanding for the molecular pathogenesis of NEC covered in this review

Necrotizing enterocolitis (NEC) is a devastating gastrointestinal disease which usually afflicts premature neonates [1, 2], however, less commonly it may also occur in full-term infants. NEC classically develops following the initiation of feeding of the predisposed newborn, and progresses from a localized gastrointestinal infection to overwhelming inflammation, pneumatosis, intestinal perforation, sepsis, and even death. As our understanding of NEC has grown, there have been advances in both therapeutic and prophylactic management strategies. This has resulted in a modest improvement in the survival rate of patients with NEC [3]. Despite significant strides in our knowledge of the pathogenesis of NEC, due to the complex and multifactorial nature of this disease, there remains gaps in our understanding. Recent advances in molecular biology have been used by investigators to study NEC. These techniques have led to new insights and these will be discussed in this review (Fig. 1).

### Current understanding of the pathogenies of NEC

The greatest risk factors for NEC are prematurity, abnormal bacterial colonization, ischemia, and formula-feeding [4–6]. In recent years, data supports the theory that immaturity of the immune system and an imbalance of inflammatory mediators play a significant role in susceptibility to NEC [7–9], and most likely contribute to the permeability, ischemic injury and intestinal barrier breakdown. Currently, there is no cure for NEC and the most effective treatments are a combination of bowel rest, antibiotics, and surgical intervention in severe cases of intestinal perforation. Patient outcomes are variable and range from survivors with minimal sequalae [10] to the challenge of life-long complications such as specific nutritional intolerances, intestinal stricture, short gut syndrome, malnutrition, and associated impairments of the immune system and neurodevelopment [6, 11–13].

To investigate NEC, a number of cell culture, organoid, and animal models have been established [14–18]. Unfortunately, there is no ideal model that perfectly mimics the pathogenesis of human NEC and each of the working models have their own unique limitations. Although models of NEC are not uniform, the majority involve induction with a combination of bacteria or their products with hypoxia and/or hypothermia or ischemia. Different models may consequently exhibit variable features of the disease as it is studied at the molecular level. Additionally, the degree of NEC severity is significantly different from that of human NEC [14]. In this review we will summarize the recent findings from a variety of animal and cellular models that contribute to the pathogenesis and progression of NEC.

### Innate neonatal risk factors

#### Immune system and inflammatory mediators

The immune system provides defense against invading pathogens. This is dependent upon two separate but closely related systems: the innate immune system (non-specific) which includes neutrophils, macrophages, and dendritic cells, and the adaptive immune system (specific) which involves T cells, B cells, and others [19, 20]. The immune system of infants is different from adults, both with regards to its content and the compartmentalization of its components. These differences are postulated to result in limited function and altered response to gut pathogens [21]. Furthermore, infants born prematurely may have significantly reduced maternal-transferred adaptive immune defense, resulting in immature intestinal epithelial cells that are vulnerable to bacterial invasion, inflammation, and intestinal ischemia [9, 19, 20].

Neutrophils are critical components of the innate immune cell population, and exit the circulation system to reach the affected tissues only when the tissues are damaged or infected with various infectious agents, including pathogens, degranulated antimicrobial peptides, and enzymes [19]. They protect the host through a defense response and exert their function by formation and release of neutrophil extracellular traps (NETs), but excessive neutrophil activity causes tissue inflammation and damage [22, 23]. NETs are web-like structures containing enriched antimicrobial protein granules and neutrophil nuclear DNAs, and have a similar function as neutrophils to destroy invading microorganisms. Both neutrophils and NETs seem to function as a two-sided sword: they will play a positive role in multiple pathophysiological mechanisms when they are at the right site at the right time, but can also contribute to complications when NETs are overly abundant or their clearance is delayed [22–25].

It has been reported that depletion of neutrophils and macrophages from the lamina propria results in exacerbation of NEC in *Cronobacter sakazakii* (CS)-treated mice compared to wild type (wt) mice, significantly increasing bacterial load, enhancing proinflammatory cytokines and tissue injury, increasing enterocyte apoptosis, and decreasing mouse survival rate [26]. Similar results were reported by *Chaaban* et al. 2020, in the case of NETs inhibition [27]. In this report, serum samples from patients with NEC (≥ stage II) exhibited significantly decreased total white blood cells and neutrophils but elevated circulating nucleosomes (histone-DNA complexes, marker for NET release). Neutrophil activation and abundant NET formation (detected by neutrophil elastase (NE) and citrullinated histone H4 (H4Cit3)) was seen in representative ileal NEC tissue.

Despite compelling evidence as to neutrophil activation in NEC, there are conflicting data regarding the role of NET inhibition in models of NEC. For example, the inhibition of NET formation with chloramidine in the ileum of an experimental NEC mouse (CD-1 mouse) led to higher bacterial translocation/defect in bacterial clearance and up-regulation of systemic proinflammatory cytokine production. No difference in intestinal injury was seen, but mortality was increased in chloramidine-treated NEC compared to NEC group. Furthermore, the decreased total white blood cells, neutrophils, and platelets in NEC was not affected by chloramidine treatment [27]. It has been reported that mice bearing ELANE (gene encoding NE)-knockout, resulting in a phenotype that does not produce NE, are protected from NEC. They also have a higher survival rate with no or little microscopic intestinal injury. Markers of neutrophil activation (NE and MPO (myeloperoxidase)) and NET formation (H3cit) were not detected by immunofluorescence staining in the intestinal tissue of these animals [14]. Similarly, studies with human NEC samples and experimental NEC mouse model demonstrated that markers of neutrophil activation (NE and MPO) and NETosis (H3cit, cfDNA (cell free DNA) and DNase), apoptosis, general inflammation (TLR4, toll-like receptor 4) and complement activation (complement component C5a) were significantly increased. NET inhibition by daily subcutaneous injection of protein arginine deiminase 4 in NEC mice resulted in significantly reduced NETs production, tissue damage, inflammation and mortality [28]. These reports indicate the important roles of neutrophils and NETs in NEC pathogenesis. The variation in these results might largely be attributed to multiple factors, including differences of individual human samples with varied ages and NEC severities, the genetic background and age of the mouse models at the induction of NEC, and the NEC induction approaches as well as the assessment time windows.

Macrophages are another crucial component of the innate immune system. Total macrophages (marker CD68 (cluster of differentiation)) and M1 macrophages (detected by iNOS (inducible nitric oxide synthase) staining) were increased in the intestinal tissue of infants with NEC, and TNF-α (tumor necrosis factor-alpha) secreted by M1 macrophages was significantly increased but c-kit (receptor tyrosine kinase) expression was significantly decreased in NEC infants [29]. Similar results were mirrored in a NEC mouse model [29]. Inhibition of macrophages by intraperitoneal injection of liposomal clodronate reduced NEC tissue injury and TNF-α expression, but increased c-kit expression. Further analysis indicated that there are 11 codes in the c-kit 5’-UTR sequence matching to 21 codes in miR (microRNA)-222 sequence, suggesting c-kit is a target gene of miR-222; luciferase assay demonstrated the decreased c-kit expression by miR-222, indicating a direct interaction between miR-222 and c-kit. In vitro experiment using 293 T cells transfected with miR-222 mimics demonstrated that TNF-α significantly suppressed c-kit expression and increased miR-222 expression [29].

CD4^+^ T cells are essential for immune homeostasis, they are tissue-specific and persistent [30], and present abundantly in infant intestine. CD4^+^ T effector memory (Tem) cells, which produce TNF-α, are present very early in fetal intestines from the end of the first trimester [31]. At 13 weeks of gestation, CD4^+^ T cells are already detected in the intestinal epithelium, and the intestinal CD4^+^ T cells are almost all αβ T cells. Their numbers further increase with age. Co-culture of fetal CD4^+^ Tem cells with intestinal stem cell (ISCs) show that low numbers of fetal CD4^+^ Tem cells supported ISC growth, but high concentration of CD4^+^ Tem cells impaired ISC development. This effect was mediated by TNF-α. NEC infants have increased numbers of CD4^+^ Tem cells in the epithelium and lamina propria of NEC-affected tissues, up-regulated TNF-α expression, and TNF-α-induced downstream gene expression, but absent IL-10 (interleukin-10) production, indicating intestinal inflammation occurs in preterm infants with exposure to antigens [31].

FOXP3 (forkhead box P3) T regulatory cells (Treg) are suppressor T cells and are essential for early intestinal immune homeostasis. The ratio of Treg to effector T cells are postulated to play an important role in the pathogenesis of NEC. Flow cytometry analysis of lamina propria T cell populations from 18 NEC and 30 non-NEC, preterm infant ileal samples indicated that Treg is abundant in NEC infants, but the ratios of Treg to CD4 and Treg to CD8 cells were significantly lower in NEC group than non-NEC infants. This was independent of the CD4 T cells and CD45RO (T cell memory) T cells, although they are also lower in NEC patients. The expression of IL-2 and TGF-β (transforming growth factor β) was unchanged, but levels of IL-1β, -6, -8, and -10, MMP3 (matrix metalloproteinase 3), MMP9, and TNF-α were increased, supporting the hypothesis that cytokines have an inhibitory effect on Treg development, and that increased inflammatory cytokine expression results in decreased Treg, thus contributing to the development of NEC [32]. Similar reports indicated that peripheral blood in NEC infants presented with significantly lower Treg cells (CD4^+^CD25^+^/^hi^FOX3^+^) and reduced suppressive activity, they also had lower IL-10^+^ and TGF-β^+^CD4^+^ T cells, but higher IL-17^+^CD4^+^ T cells [33].

Toll-like receptors play an essential role in activation of the innate and adaptive immune systems. Surgical intestinal specimens from infants with NEC and mice with experimental NEC, exhibit significantly higher TLR4 (known to recognize pathogen and activate innate immunity) signaling activity [34]. Mouse pups with NEC expressing an inhibitory mutation of TLR4 showed a marked reduction in the incidence and severity of NEC, and a significant reduction in apoptosis compared to mice expressing wildtype TLR4. TLR4 inactivation increased Intestinal Epithelial Cell Line 6 (IEC-6) migration and proliferation, decreased intestinal apoptosis, and increased restitution and proliferation after mucosal injury [35]. Furthermore, TLR4 co-immunoprecipitated with focal adhesion kinase (FAK) (FAK is required for and controls cell migration) and inhibition of FAK lead to restoration of enterocyte migration after TLR4 activation, suggesting the regulating role of TLR4-FAK association on intestinal healing [34]. Another study also demonstrated that TLR4 knockout protected NEC-induced tissue damage in mice, decreased the elevated expression of caspase-8, phosphorylated necroptotic protein RIPK (receptor-interacting protein kinase) 1 and 3, and their substrate pMLKL (phosphorylated mixed-lineage kinase domain-like protein), but significantly increased survival rate [35]. Hackam et al.2019 [36] summarized these findings from their series of studies and others’ works on the relationship of TLR4 and NEC, in which TLR4 is required for normal gut development in mice. Therefore, it is not surprising that TLR4 expression is significantly higher in the premature intestine of mice and humans compared with the full-term gut due to this normal developmental pattern of expression [37]. Moreover, higher levels of TLR4 in the premature gut and its activation in the postnatal gut lead to intestinal ischemia, barrier compromise, and reduced intestinal restitution, which in turn leads to NEC [36, 37].

### Epigenetic alterations and regulatory elements

#### Epigenetic alterations

Epigenetic alterations or modification describe changes in gene function and regulation without actual changes to the DNA structure and can be frequently linked to pathogenesis of certain diseases. These alterations include DNA methylation, chromatin histone modification and non-coding RNAs [38]. Microarray-based DNA modification profiling revealed that there are more than 200,000 regions with differential DNA modification between human fetal immature intestinal epithelial cells (H4) and human adult mature intestinal epithelial cells (NCM460). Exposure to probiotic and pathogenic bacteria induced different epigenetic effects in immature intestinal epithelial cells when compared to mature intestinal cells, creating increased sensitivity to these bacteria [39]. To better understand the role of alterations in the fetal intestine, epigenetic modifications were studied in an intestinal cell line as well as an in vivo mouse model in response to steroid administration. H4 cells were treated with dexamethasone (a synthetic glucocorticoid) for 48 h and revealed that 174 regions with differential DNA modifications compared to untreated cells. For further investigation in vivo, prenatal exposure of a mouse model to dexamethasone resulted in alterations of DNA methylation profiles and changes of gene expression in the TLR-, and TJ (tight junction)-associated signaling pathways in the ileal tissue of two-week-old offspring. Furthermore, epigenetic changes are associated with alteration in the gut microbiome in offspring [39].

Epigenetic DNA methylation often occurs in cytosine-guanine dinucleotides (CpGs) in many regions of a gene, and has a significant impact on gene regulation and function. DNA was isolated from stool samples of 24 preterm infants with NEC and 45 age-matched controls and was used to measure epigenetic alteration in the form of DNA methylation. The methylation pattern was investigated at various time intervals around the development of NEC: long before NEC (develop NEC after the first 28 days), short time before NEC onset (one week before onset) and after NEC. Pyrosequencing was then performed on these stool samples to elucidate the specific epigenetic alterations of several genes previously linked to the pathogenesis of NEC. TLR4 CpG2 methylation, VEGFA (vascular endothelial growth factor isoform A) CpG3 methylation, and DEFA5 (defensin α5) CpG1 methylation were present in NEC infants, but differed in expression based on the time of NEC onset. TLR4 methylation was only increased in prior to NEC, suggesting a down-regulation of TLR4, possibly contributing to an inadequate inflammatory response. Hypermethylation of VEGFA and DEFA5 (genes contributing to the microvasculature and perfusion of intestinal epithelium) were found in infants at a short time before NEC and after NEC compared to long before NEC group. This suggests that alterations in these genes may result in underdevelopments of intestinal vasculature and perfusion, contributing to the infants increased susceptibility to NEC [40].

High-throughput whole-genome bisulfite sequencing for epithelial methylation signatures using 7 ileal samples of surgical NEC and 11 from non-NEC revealed that global difference of CpG methylation (2785 sites with methylation rate of at least 10%). Surgical NEC ileum and non-NEC ileum in exons, introns, intergenic regions, CpG islands and enhancers in which NEC samples have higher methylation levels, except CpG island shores and promoters which have little to no difference in the methylation level. Gene function analysis of the promoter methylated genes indicated that these genes are involved in signal pathways of IL-17, CD40, induction of apoptosis, and granulocyte adhesion and diapedesis. There are 652 significant differentially methylated single CpG sites between NEC and non-NEC ileum with 30 of them showed most highly significant differences. Further analysis of NEC-specific transcriptional changes using RNA sequencing identified 649 mRNAs (75.7%) that were elevated in expression in surgical NEC ileum compared to non-NEC control and 208 mRNAs (24.3%) showed reduced expression, indicating a clear correlation between the gene expression and DNA methylation. Functional pathway analysis of differentially expressed transcripts indicated they are involved in acute phase response signaling, granulocyte adhesion, diapedesis, and hepatic fibrosis/hepatic stellate activation [41].

#### Regulatory RNAs

Long non-coding RNAs (lncRNAs, also termed as competing endogenous (ce) RNAs, > 200 bp) and microRNAs (miRNAs, miRs, a class of small single-stranded non-coding RNAs, ~ 22 bp) have been shown to play important roles in cellular physiological activities through regulation of gene expression. A number of studies have shown that lncRNAs regulate the expression of TJ proteins, which is supportive of their role in the regulation of intestinal epithelial barrier function [42–44]. Deep Illumina sequencing of rat ileum with and without NEC, identified 1820 lncRNAs (among differentially expressed 16,518 lncRNAs), 118 miRNAs and 929 mRNAs that were differentially expressed in NEC when compared to controls. Gene Ontology (GO) and Kyoto Encyclopedia of Genes and Genomes (KEGG) pathway analysis showed that target genes of lncRNAs, miRNAs and mRNAs are highly associated with the regulation of several cellular processes: TLR4 signaling pathway, PI3K regulatory subunit binding, apoptosis, regulation of NF-κB activity, chemokine-mediated signaling pathway, as well as mTOR (mammalian target of rapamycin) and Notch signaling pathway [45]. Furthermore, construction of lncRNA-miRNA interaction network, in which mRNAs with the same miRNA-binding sequence as lncRNAs for regulating target mRNAs, revealed that there are 129 lncRNA nodes and 93 miRNA nodes that are differentially regulated in response to NEC. Analysis of four particular lncRNAs demonstrated that each has the ability to bind to one corresponding miRNA; all of which are implicated in the development of intestinal disease. Verification of the expression of these four lncRNAs by qRT-PCR confirmed the results obtained from deep Illumina sequencing and was shown by GO and KEGG analysis to be involved in regulation of TLR4, mTOR and Notch signaling pathways [45]. In other studies, RNA sequencing was performed on intestinal tissue from patients with Bell’s stage III NEC. These samples were compared with control samples, and showed that 4202 lncRNAs and 7860 mRNAs were significantly differentially expressed between these two groups. GO and KEGG analysis identified that the differentially expressed lncRNA host genes and mRNAs were closely associated with inflammatory responses including, TLR4, PPAR (peroxisome proliferator-activated receptor), PI3k-Akt, HIF-1 (hypoxia-inducible factor 1), and TGF-β signaling pathways. The IncRNA-mRNA co-expression network further identified some of the lncRNAs that are closely associated with NEC [46].

It has also been hypothesized that particular miRNAs play a protective role in NEC. Namely, miRNA-146a-5p has been reported as a valuable anti-inflammatory factor and its expression is increased in macrophages associated with NEC and other intestinal inflammatory disorders [47]. NLRP3 (nucleotide-binding domain and leucine-rich repeat-containing protein 3) inflammasome and its downstream factors IL-1β and IL-18 are also increased in NEC tissue. Overexpression of miRNA-146a-5p was shown to inhibit the downstream effects of the NLPR3 inflammasome, ameliorated intestinal injury, and lead to increased survival of mice with NEC [47].

miRNA microarray profiles of 10 human NEC (Bell’s stage III) tissues revealed distinct and significant differences between NEC and controls, analysis of the correlation between miRNAs and their mRNA targets suggested the interaction of dysregulated miRNA/mRNA pairs in NEC with TLR4 (miR-31, -451,-203,-4793-3p), NF-κB2 (miR-203), FOXA1 (miR-21-3p, miR-431, and miR-1290) and HIF1α (miR-31) and downstream pathways of angiogenesis, hypoxia/oxidative stress, inflammation and muscle contraction [48]. Another study showed that miR-431 was significantly higher and FOXA1 was significantly lower in 10 NEC infants (stage III) compared to the controls, and FOXA1 was predicted as a top target of miR-431. In vitro validation of direct binding of miR-431 and 3’-UTR of FOXA1 in HEK293 and caco-2 cells with luciferase as a reporter showed that luciferase activity of the cells transfected with the FOXA1 wt 3’-UTR reporter was significantly inhibited by miR-431 mimics. Overexpression of miR-431 in caco-2 cells suppressed FOXA1 expression both in mRNA and protein levels; Further detection of the potential downstream target genes of miR-431 in caco-2 cells overexpressing miR-431 indicated that the levels of ESRRG (estrogen related receptor gamma), FOXA1, and HNF4A (hepatocyte nuclear factor alpha) were significantly reduced but LGR5 (leucine rich repeat containing G protein-coupled receptor 5), NF-κB2, PLA2G2A (phospholipase A2 Group IIA), PRKCZ (protein kinase C, zeta), IL-6 and TNF were significantly increased. Their expression was sustained when co-stimulated with LPS (lipopolysaccharide) or LTA (lipoteichoic acid). However, the expression of IL-8 and IL-10 was not affected by miR-431 alone, but was significantly increased in the presence of LPS (IL-8) or LTA (IL-8 and IL-10). Moreover, overexpression of miR-431 in Caco-2 cells significantly decreased cell proliferation and increased apoptosis. Expression was not altered in the presence of LPS or LTA. Finally, regulatory network of the miR-431-FOXA1 axis was constructed with MetaCore software and showed potential interaction with TLR2 and TLR4 to regulate genes associated with cell apoptosis and proliferation (LGR5, ESRRG), inflammation (IL-6, -8, -10, TNF, PLA2G2A) and intestinal epithelial tight junction (HNF4A, PRKCZ) [49].

### Single nucleotide polymorphisms (SNPs)

There are abundant SNPs in the human genome, but most of these genetic variations do not cause changes in gene expression, protein structure or function [50]. However, some SNPs are associated with increased or decreased risk of NEC. Investigation of a large group of preterm newborns (358 cases with 26 cases of NEC ≥ stage II) for the association of SNPs with risk of NEC in VEGF C-2578A (rs699947), IL-18 C-607A (rs1946518), and IL-4Rα (IL-4 receptor α-chain) A-1902G (rs1801275) revealed that all three SNPs were not significantly different from infants with or without NEC under various genetic models, but the G-allele of the IL-4Rα A-1902G SNP was significantly negative association with the outcome NEC or death [51]. Using the SEQUENOM Mass Array platform assay, data from 30 NEC patients and 80 controls in Chinese Han population indicated that the VEGFA SNPs rs699947 and rs833061 were low in plasma and are associated with high risk of NEC [52]. Investigation of 751 patients with extremely low birth weight (30 had surgical NEC) using genome-wide association study tool PLINK for the link between genetic variations and increased risk of surgical NEC (stage III) revealed that 261 SNPs are allelic differences in NEC vs. no NEC, 35 of that are significantly different. A strong association between a cluster of SNPs with NEC occurred on chromosome 8 at the location 8q23.3, spanning 43 kb, termed NECRISK cluster for this region, NEC has the highest significance of association; there are two other the most significant clusters of SNPs occurred on chromosome 14 and 11. Silicon analysis and RNA sequencing identified that the nearest potential novel transcript to the NECRISK region is 96% homologous to the long interspersed element-1 retrotransposon, and its activation may result in gene interruptions or effects of gene expression in the vicinity of integration, or result in structural variants of chromosome on the incidence of NEC; The second most significant cluster (on chromosome 14) associates with the ADCY4 (adenylate cyclase 4) and LTB4R (leukotriene B4 receptor) genes, which are involved in regulating signaling in epithelial cells and inflammation, and may be associated with pathogenesis of NEC, if not directly; and the third (on chromosome 11) corresponds to the neurogranin gene, which plays a role in IL-2-dependent survival of T-cells, indicating a role involves in immune/inflammatory signaling [53].

The SNPs of the transmembrane protease serine 6 gene (TMPRSS6) rs855791, and the hemochromatosis gene (HFE) rs1800562 and rs1799945 are known to be associated with increased serum iron levels in adults. 11,166 infants were completely genotyped for all three SNPs. The data revealed that high serum iron levels and low intestinal levels due to rs855791 genotype G-allele were associated with a significantly reduced risk of NEC surgery, but the A-allele had low iron uptake and higher intestinal iron level leading to a higher risk of NEC surgery [54]. This correlation of low iron uptake with high risk of NEC can also explain the phenomenon that formula feeding of preterm infants had an increased risk of NEC because of the low lactoferrin content and higher iron content. However, no significant differences were seen in the infants with rs855791 A-allele (low iron uptake) compared to the infants with rs855791 G-allele (high iron uptake) following 5 years follow up outcome from 946 infants. This finding supported the fact that oral iron intake in preterm infants is not associated with NEC [54].

It was reported that examination of 31 SNPs in 9 different DUSP (dual specificity phosphatase, a key suppressor of mitogen-activated protein kinase (MAPK) pathways) genes using Agena Mass Array assay demonstrated that the presence of the rs704074 SNP was associated with greatly decreased risk of developing NEC and remarkably decreased surgical NEC in survey of 50 (≥ stage II) cases [55].

### Microvasculature

Differences in the premature gut microvascular system have been implicated in the pathogenesis of NEC. Indeed, the underdeveloped intestinal microvasculature in premature neonates of murine NEC models is unable to support the metabolic needs for neonatal growth, thus resulting in intestinal ischemia and necrosis [56]. The microcirculation has been suggested to significantly contribute to the development of NEC in a rat model. In this model, pups with NEC exhibited small arterioles with an altered arteriolar flow pattern, and elevated levels of inflammatory mediators including TLR4, IL-1β and HMGB1 (high-mobility group box protein 1) compared to controls [57].

Decreased expression of VEGFA was detected in human NEC [58, 59], prior to detectable intestinal injury in murine NEC [59]. Overexpression of VEGF in experimental rat alleviated villous atrophy and tissue edema, decreased oxidative stress and the expression of IL-6, TNF-α and caspase 3, but increased MPO level [60]. Similarly, the expression of VEGFR2 (VEGF receptor 2) was reduced in NEC mice. Inhibition of VEGFR2 kinase activity increased the incidence and severity of NEC, and decreased the density of intestinal vascular network [59, 61]. Moreover, prenatal inflammation increased susceptibility to NEC, significantly decreased intestinal microvascular density, and downregulated intestinal VEGF and VEGFR2 signaling [62]. In neonatal mice, stabilizing HIF-1α (the master regulator of VEGF) with dimethyloxalylglycine (DMOG) resulted in decreased incidence of severe NEC, increased intestinal VEGF expression, and increased villus endothelial and epithelial proliferation, these effects are VEGFR2-dependent [61]. Furthermore, decreased intestinal microvascular density, reduced VEGF and VEGFR2 protein expression and increased the incidence of severe NEC by postnatal TNF administration in NEC mice were ameliorated by DMOG, demonstrating the cause of NEC is through a TNF-dependent deficiency of intestinal microvascular development [62].

A very recent report demonstrated that the highest expression of HIF-1α presented in the most damaged human NEC tissues accompanied by complete absence of microvasculature compared to the less affected/not-affected ileum, indicating NEC is associated with hypoxia, reduced endothelial cell number and microvascular vessels [63]. In experimental mouse NEC, intestinal damage was first detected at P6, and the most significant morphological changes and inflammation starting at P7. Application of RIC (remote ischemic conditioning) at P5 (minimal damage or not present) or P6 significantly improved intestinal morphology, reducing inflammation and survival of NEC pups, but has no protection on injury if application of RIC at P7. Application of RIC on eNOS (enzyme nitric oxide synthase) knockout pups at the same age was unable to result in the same effects, indicating RIC-mediated intestinal protection in NEC is dependent on endothelium-mediated vasodilation. Further investigation demonstrated that RIC action through preserving intestinal perfusion and integrity of the villi microvasculature by promoting vasodilation and enhanced blood flow in the immature intestine [63]. These findings are supportive of the microvasculature’s critical role in the development of NEC, and that timing and expression levels of VEGF and VEGFR2 are key events in the signaling pathways that contribute to the development of NEC. However, additional supporting evidence to link alterations in microcirculation system to NEC are still needed.

## Conclusion

Current understanding of the pathogenesis of necrotizing enterocolitis involves a multifactorial molecular framework that continues to be expanded through the study of its mechanisms in animal and cellular models. Emphasis is placed on factors that are a consequence of an immature immune system of the affected premature neonate. This leads to impaired gut barrier that is more susceptible to infection. The immune response and signaling that ensues after bacterial infection are dysregulated and result in overwhelming inflammation that manifests in intestinal injury seen in NEC. Above are factors inherent to the immature immune system characteristic of the premature neonate. In addition, certain epigenetic modifications and regulatory elements have been identified that may further make an affected neonate more susceptible to NEC. Finally, underdeveloped microvasculature and its underlying molecular regulators are implicated in development of NEC. Further investigation into the described molecular signaling pathways and newly identified pathogenic mechanisms continue to characterize NEC and add to its complexity, with expectation that a better understanding of the molecular basis for the disease will translate into development of potentially life-saving and preventative therapy.

## Data Availability

Please see the References section to access all citations for studies used to support this review.

## Abbreviations

ADCY4: Adenylate cyclase 4
CD: Cluster of differentiation
cfDNA: Cell free DNA
c-kit: Receptor tyrosine kinase
CpGs: Cytosine-guanine dinucleotides
DEFA5: Defensin α5
DMOG: Dimethyloxalylglycine
DUSP: Dual specificity phosphatase
eNOS: Enzyme nitric oxide synthase
ESRRG: Estrogen related receptor gamma
FAK: Focal adhesion kinase
FOXP3: Forkhead box 3
GO: Gene Ontology
H4Cit3: Citrullinated histone H4
HFE: Hemochromatosis gene, human homeostatic iron regulator protein
HIF-1: Hypoxia-inducible factor-1
HMGB1: High-mobility group box protein 1
HNF4A: Hepatocyte nuclear factor alpha
IL-10: Interleukin-10
iNOS: Inducible nitric oxide synthase
ISCs: Intestinal stem cells
KEGG: Kyoto Encyclopedia of Genes and Genome
LGR5: Leucine rich repeat containing G protein-coupled receptor 5
lncRNAs: Long noncoding RNAs
LPS: Lipopolysaccharide
LTA: Lipoteichoic acid
LTB4R: Leukotriene B4 receptor
MAPK: Mitogen-activated protein kinase
miR: MicroRNA
MLKL: Phosphorylated mixed-lineage kinase domain-like protein
MMP3: Matrix metalloproteinase 3
MPO: Myeloperoxidase
mTOR: Mammalian target of Rapamycin
NE: Neutrophil elastase
NEC: Necrotizing enterocolitis
NETs: Neutrophil extracellular traps
NLRP3: Nucleotide-binding domain and leucine-rich repeat-containing protein 3
PLA2G2A: Phospholipase A2 Group IIA
PPAR: Peroxisome proliferator-activated receptor
PRKCZ: Protein kinase C, zeta
RIC: Remote ischemic conditioning
RIPK: Receptor-interacting protein kinase
SNPs: Single nucleotide polymorphisms
Tem: T effector memory
TGF-β: Transforming growth factor β
TJ: Tight junction
TLR4: Toll-like receptor 4
TMPRSS6: Transmembrane protease serine 6 gene
TNF-α: Tumor necrosis factor α
Treg: T regulatory cells
VEGFA: Vascular endothelial growth factor isoform A
VEGFR2: VEGF receptor 2
Wt: Wild type
